# Stress-first single photon emission computed myocardial perfusion imaging

**Published:** 2016-11-01

**Authors:** C I Aquino, M Scarano, F Squame, G Casaburi, S L Nori, L Pace

**Affiliations:** 1 Dipartimento di Medicina, Chirurgia e Odontoiatria “Scuola Medica Salernitana”, Università degli Studi di Salerno, Italy; 2 A.O.U. S. Giovanni di Dio e Ruggi d’Aragona, Salerno, Italy

**Keywords:** Myocardial Perfusion Imaging, Coronary Artery Disease, Radiation Dose, Stress-First

## Abstract

**Background:**

Myocardial perfusion imaging (MPI) with single photon emission tomography (SPET) is widely used in coronary artery disease evaluation. Recently major dosimetric concerns have arisen. The aim of this study was to evaluate if a pre-test scoring system could predict the results of stress SPET MPI, thus avoiding two radionuclide injections.

**Methods:**

All consecutive patients (n=309) undergoing SPET MPI during the first 6 months of 2014 constituted the study group. The scoring system is based on these characteristics: age >65 years (1 point), diabetes (2 points), typical chest pain (2 points), congestive heart failure (3 points), abnormal ECG (4 points), male gender (4 points), and documented previous CAD (5 points). The patients were divided on the basis of the prediction score into 3 classes of risk for an abnormal stress-first protocol.

**Results:**

An abnormal stress SPET MPI was present in 7/31 patients (23%) with a low risk score, in 24/90 (27%) with an intermediate score risk, and in 124/188 (66%) with an high score risk. ROC curve analysis showed good prediction of abnormal stress MPI.

**Conclusions:**

Our results suggest an appropriate use of a pre-test clinical prediction formula of abnormal stress MPI in a routine clinical setting.

## INTRODUCTION

Single photon emission tomography myocardial perfusion imaging (SPET-MPI) is one of the most used and most accurate non invasive method of evaluation of patients with coronary artery disease. In the last 20 years, however, a significant reduction of abnormal findings on SPET-MPI has been observed. Actually, Rozanski et al. reported a gradual decline in the frequency of abnormal perfusion studies from 41% in 1991 to 9% in 2009 1. Thus, concerns have arisen on over utilization of SPET-MPI, particularly in low risk patients 2. It should be noted that the acquisition protocol still in use have been developed several years ago. Since then major concerns on radiation exposure, in the last few years a 6-fold increase in background radiation from medical imaging has been observed 3; moreover, health care costs have arisen. The routinely used protocol of SPET-MPI is based on two administration of the radiotracer: one at rest and one during stress. Since few abnormal studies are expected to be found in routine applications, a reasonable way to reduce both radiation dose and costs could be to avoid the rest injection of the radiotracer and thus the rest SPET-MPI acquisition if stress SPET-MPI shows normal myocardial perfusion. A strategy of stress-first SPET-MPI, leading to stress-only if images are normal, has been proposed over two decades ago 4, and many authors as well as Scientific Societies enforced it because of reduced radiation exposure and costs with improved laboratory efficiency 5–11. A stress-only approach would reduce radiation dose to less than 30%–60% and costs would be decreased because of the reduction of examination time (<90 minute instead of 3–5 hours) leading to a reduced use of the medical equipment and an increase in the number of patients examined daily 12, 13.

Actually, not all people can be tested with the stress-first technique. Main criteria of eligibility are: presence of symptoms in a patient with a low likelihood of ischemia, no history of documented myocardial infarction and/or revascularization (PCI and/or CABG), a recent normal functional or anatomic study 14, 15. Recently, Duvall et al 14 proposed a pre-test scoring system based on clinical variables to accurately identify patients who can successfully undergo a stress-first imaging protocol without the need for rest imaging. Thus, the aim of this study is to evaluate in a routine setting if the pre-test scoring proposed by Duvall et al 14 could predict an abnormal stress SPET-MPI.

## METHODS

All consecutive patients (n=309) undergoing SPET-MPI during the first 6 months of 2014 in the Nuclear Medicine Department of San Giovanni di Dio e Ruggi D’Aragona University Hospital constituted the study group. None of the patients was in the Emergency Department and none of them had an available recent (i.e. < 3 months) coronary angiography. Demographic and stress test variables at the time of SPET-MPI were collected for all patients (Table I). Demographic variables recorded were age, gender, height, weight. Clinical variables collected were chest pain, shortness of breath, diabetes, hypertension, hyperlipidemia, smoking, family history of CAD, peripheral vascular disease, cerebrovascular disease, congestive heart failure, documented CAD (which included known CAD by diagnostic testing or patient history, history of myocardial infarction, history of revascularization), abnormal ECG, previous normal stress MPI, previous normal coronary angiography, congestive heart failure, pulmonary hypertension, and stressor used.

The scoring system is based on the following parameters, linked with a specific score: age >65 years (1 point), diabetes (2 points), typical chest pain (2 points), congestive heart failure (3 points), abnormal ECG (4 points), male gender (4 points), and documented CAD (5 points) 14. According to the proposed scoring model 14, all the patients were divided into 3 classes of risk for an abnormal stress SPET-MPI: low risk (<5), intermediate risk (≥5 <10) and high risk (≥10).

SPET-MPI was performed according to standard imaging protocol as endorsed by ASNC 16,17. A rest-stress or stress-rest imaging sequence was employed using Tc-99m sestamibi. All patients underwent physical exercises. SPET-MPI was performed using a dual head camera (CardioMD, Philips), equipped with a high resolution collimator, stop and shoot acquisition with 64 steps, a 180°arc from right anterior oblique to left anterior oblique, a 64 × 64 × 16 matrix, using an iterative reconstruction algorithm (Astonish). Image acquisition began 30–60 minutes after radiotracer injection. A 17-segment model was applied for semi quantitative visual analysis of SPET-MPI images. For each myocardial segment a 5-point scoring system was used: 0= normal perfusion, 1= mild reduction in counts (not definitely abnormal), 2= moderate reduction in counts (definitely abnormal), 3= severe reduction in counts, 4= absent uptake. In addition to individual scores, the summed scores were calculated. A summed stress scores (SSS) was obtained by adding together the stress scores of all the segments and the summed rest score (SRS) by adding together the resting scores of all the segments. Stress SPET-MPI was considered abnormal with a SSS >3.

Previously unpublished data obtained in our laboratory in 95 patients showed an ICC= 0.98 for intraobserver reproducibility and and ICC=0.97 for interobsever reproducibility (p<0.001 for both) of visual analysis.

MedCalc Statistical Software version 13.1.2 was used for statistical analysis (MedCalc Software bvba, Ostend, Belgium; http://www.medcalc.org; 2014). All data are expressed as mean + 1 standard deviation or as percentage, as appropriate. Receiver operating curve (ROC) analysis was used to assess the accuracy of the predictive model and to assess the accuracy of the predictive model and to determine the optimal cutoff by using the Youden index^18^. A p value < 0.05 was considered significant.

## RESULTS

**Fig. 1:**
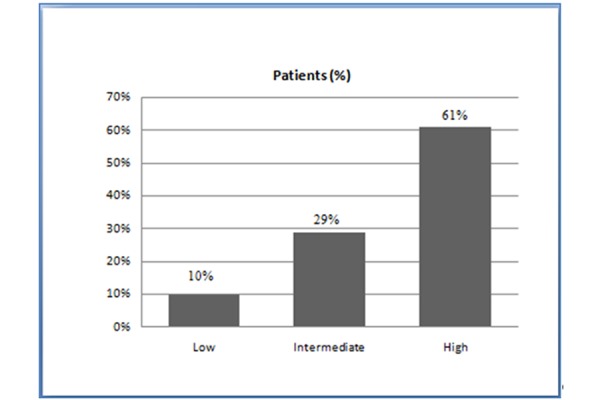
Distribution of patients in the risk groups based on prediction score.

**Fig. 2:**
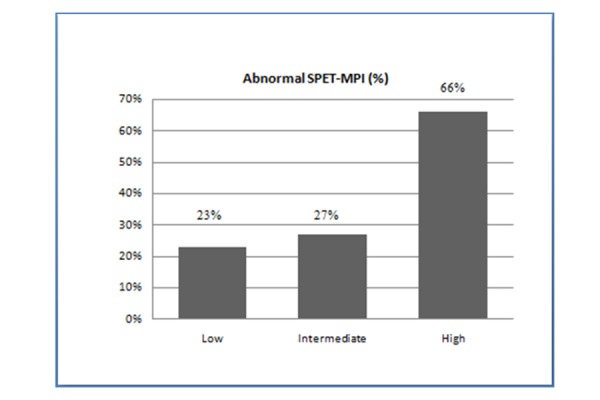
Observed abnormal stress MPI in predicted risk group.

Table 1 shows the clinical and demographic variables of the patients included in the study. Of the 309 patients analyzed, 31 (10%) presented a low score risk, 90 (29%) had an intermediate score risk, and 188 (61%) showed a high score risk (Figure 1). Seven (23%) of the 31 patients in the low risk group had an abnormal stress SPET-MPI, 24 (27%) of the 90 patients in the intermediate risk group showed an abnormal stress SPET-MPI, and 124 (66%) of the 188 patients in the high risk group had an abnormal stress SPET-MPI (Figure 2).

**Fig. 3:**
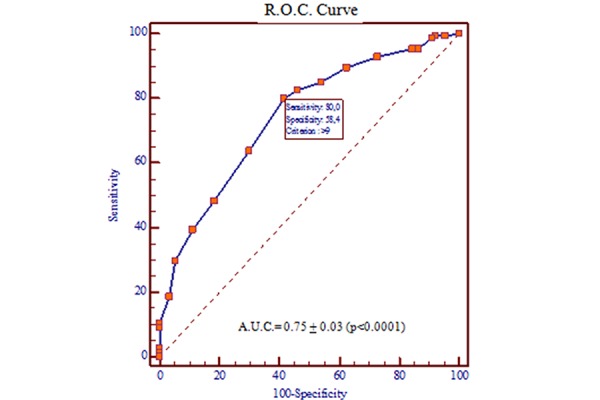
ROC curve of the stress-first prediction score.

ROC curve analysis showed good prediction of abnormal stress SPET- MPI (Figure 3) with an area under the ROC curve of 0.75. Using the optimal cutoff selected by the ROC curve analysis, sensitivity was 80% and specificity was 58%.

## DISCUSSION

In the present study, the majority of the patients with low or intermediate risk of abnormal stress SPET-MPI (90/121 patients,74%) would not need rest images as their stress perfusion images were interpreted as normal. These results are comparable to those obtained by Duvall et al 14, suggesting a possible use of the proposed pre-test clinical prediction model of abnormal stress SPET-MPI in a routine clinical setting. Moreover, the prevalence of normal MPI is in the same range (60–70%) in many large published reports 12, 13, 14, 20, 21. These evidences suggest a probably redundancy of rest SPET-MPI in many patients where a normal stress study obviates the need for rest imaging, as stated by the European MPI guidelines 22.

The routine procedure adopted in many clinical nuclear medicine centers is based on two separate radiotracer injection (stress and rest) and obviously two SPET-MPI. The two injections could be performed in the same day, 2–3 hours apart, or in two separate days. The procedure requires 3 to 5 hours to be performed, when a single day protocol is adopted, or 1 to 2 hours for each day when a 2-day protocol is scheduled. Of course, two radiotracer administrations lead to a higher radiation exposure, often unnecessary 12,13. A stress-first SPET-MPI can decrease both procedure time and radioactive dose, avoiding the rest scan if the stress one is normal. All these advantages are relevant to the health care system^12^, ^13^, ^19^, ^23^. Moreover, avoiding the rest SPET-MPI when a normal stress SPET-MPI is found would not affect the clinical relevance of the study, since a low cardiac event rate is associated with a normal stress-only study, with an annualized cardiac event rate < 0.7% 19, 24.

Recently, new diagnostic imaging techniques in CAD patients have been introduced showing excellent results, namely Cardiac Computed Tomography, which has been proposed as an alternative to SPET-MPI. MPI SPECT in low-intermediate risk CAD patients optimized with stress only imaging is similar to Cardiac Computed Tomography in time to diagnosis, length of hospital stay, and cost, with improved prognostic accuracy and less radiation exposure 25. The efficacy of stress-only protocol has been evaluated in several studies including a variety of subjects: in-patients, outpatients, and the emergency department 12, 13, 20, 21, 26.

An effective use of the stress-first SPET-MPI protocol requires an appropriate selection of patients to be studied with. Criteria for selecting patients for a stress-first imaging protocol can be: no symptoms suggestive of ischemia and low to intermediate pre-test probability, no history of documented myocardial infarction and/or coronary revascularization, a history of a recent normal functional or anatomic study. A key point in stress-first protocols is the presence of the physician who should select the protocol for each patient and check the presence of any perfusion abnormality on stress SPET-MPI and thus decide to perform the rest scan. A way to limit the number of abnormal stress-first studies to be analyzed would be to perform rest-stress studies only in patients with a history of CAD or myocardial infarction who are considered “high risk”. However, defining exactly who is an “high risk” patient could be difficult. On the other hand, a predictive scoring system could help in the selection of patients with a high probability of a normal stress SPET-MPI, i.e. low risk patients. Duvall et al. 12, in particular, analyzed a large court of patients identifying a 92% success rate for the low risk group with a stress-first protocol and an area under the ROC curve of 0.82.

The pre-test scoring tool we used in the present study is able to predicts patients who have a high likelihood of successfully completing a stress-first imaging protocol without the need for rest imaging on the basis of level of risk. Actually, while 77% of patients with low-intermediate risk do have normal myocardial perfusion at stress SPET-MPI, 66% of those with high risk showed abnormal myocardial perfusion. Thus, it would be conceivable to perform a stress-first SPET-MPI protocol in patients in low or intermediate pre-test risk classes.

The finding of a similar prevalence of abnormal findings in low and intermediate risk patients clearly indicates that the model is not able to discriminate between these two classes of risk. This results is different from what reported by Duvall et al^12^, and could be due to differences in the populations studied, as we do not have patients from the Emergency Department, or to differences in acquisition methods, since we do not have attenuation correction. However, it should be noted that using the best cutoff selected by the ROC curve analysis we obtained good results in selecting patients suitable for stress-only myocardial perfusion imaging.

The present study has some limitations. The retrospective collection of data and the relatively low number of the patients could prevent from a general conclusion. Benefits of a prediction formula would be of course more relevant in a larger cohort. Moreover, the camera used in our study does not allow attenuation correction. However, the good results we obtained without attenuation correction indicate that the proposed model is quite robust and can be used in routine practice. Finally, no gated MPI has been performed. Although it is true that gated acquisition is important, the finding of normal wall motion in a myocardial segment showing a perfusion abnormality on stress image without attenuation correction does not change the perceived need for a rest study or the interpretation certainty because the stress perfusion abnormality may represent either ischemia or attenuation artifact^26^.

Applying a stress-first protocol in a routine clinical setting leads to some logistic and dosimetric consideration. Clinical and demographic characteristic of the patient must be known before data acquisition to decide the opportunity to perform a stress-first acquisition for each patient. It should be noted that all the parameters used for the score can be easily obtained from the clinical history and / or the medical record of each patient. Furthermore, decisions may be taken in advance or upon arrival of the patient in the Nuclear Medicine laboratory even by different members of the staff. A key point is the need to analyze stress images as soon as possible. This implies that the nuclear medicine physician in charge must be present in the elaboration room and read the MPI data just at the end of the data acquisition. From a dosimetric point of view, besides the dose reduction for the patients, the radiation burden is also reduced for the staff. Indeed, the clinical data collection takes place before the administration of the radiotracer and avoiding the rest injection of the radiotracer in selected patients would save the member of the staff in charge of injection a second irradiation.

In conclusion the results of the present study suggest an appropriate use of a pre-test clinical prediction formula of abnormal stress MPI in a routine clinical setting.

## Figures and Tables

**TABLE 1:** PATIENTS’ CLINICAL CHARACTERISTCS

Variables	

Age (years)	66 ± 10
Gender: Male (%)	209 (67%)
BMI (kg/m^2^)	28.1 ± 4.2
Chest pain	90 (29%)
Dyspnoea	15 (5%)
Diabetes	65 (21%)
Hypertension	191 (62%)
Hyperlipidemia	72 (23%)
Smoking at time of MPI	3 (1%)
Heart failure	31 (10%)
Documented CAD	216 (70%)
Abnormal stress MPI	155 (50%)
Positive ECG	68 (22%)
